# The effects of a music intervention during port catheter placement on anxiety and stress

**DOI:** 10.1038/s41598-021-85139-z

**Published:** 2021-03-11

**Authors:** Nora K. Schaal, Johanna Brückner, Oliver T. Wolf, Eugen Ruckhäberle, Tanja Fehm, Philip Hepp

**Affiliations:** 1 Department of Experimental Psychology, Heinrich-Heine-University, Universitätsstraße 1, 40225 Düsseldorf, Germany; 2 Department of Cognitive Psychology, Institute of Cognitive Neuroscience, Faculty of Psychology, Ruhr-University, Bochum, Germany; 3 Clinic for Gynecology and Obstetrics, Heinrich-Heine-University, Düsseldorf, Germany; 4 Clinic for Gynecology and Obstetrics, University Clinic, Augsburg, Germany; 5 Clinic for Gynecology and Obstetrics, HELIOS University Hospital Wuppertal, University Witten/Herdecke, Wuppertal, Germany

**Keywords:** Health care, Oncology

## Abstract

Studies have shown that perioperative music interventions can reduce patients’ anxiety levels. However, in small operations like port catheter surgery evidence is sparse. The present single-blinded, randomised controlled two-armed study included 84 female patients undergoing port catheter placement who were randomly assigned to either listening to music during surgery vs. no music intervention. The medical staff was blind to group allocation. On the day of the surgery anxiety and stress levels were evaluated using subjective (STAI questionnaire, visual analogue scales) and objective (vital parameters, salivary cortisol) parameters at different time points (before the surgery, at the end of the surgery and 1 h post-surgery). The music group showed significant reductions of systolic blood pressure (from 136.5 mmHg ± 26.1 to 123.3 mmHg ± 22.0, *p* = .002) and heart rate (from 75.6 bpm ± 12.3 to 73.1 bpm ± 12.2, *p* = .035) from beginning of the surgery to skin suture, whereas the control group did not. No significant effects of the music intervention on subjective anxiety measures or salivary cortisol were revealed. In sum, the study demonstrates that a music intervention during port catheter placement positively influences physiological anxiety levels, whereas no effects were revealed for subjective anxiety and salivary cortisol. Thus, music can be considered as a low cost addition in clinical routine in order to reduce patients’ heart rate and blood pressure. Future studies are encouraged to further explore the differential effects of intraoperative music interventions on physiological, endocrinological and subjective anxiety levels.

## Introduction

Many patients who undergo surgical procedures in analgosedation experience anxiety and stress^1,2^, which can have a negative influence on pain perception^3^. Therefore, it is important to examine non-pharmacological intraoperative interventions, which may be able to reduce anxiety and stress.

The efficacy of music interventions in the context of invasive procedures on stress and anxiety has been demonstrated in several studies in many areas of medicine (for reviews see^4,5^). It has been shown that music interventions lead to a reduction of subjective anxiety levels^6–8^, lower heart rate^8,9^ and blood pressure levels^6,8^, and lower levels of salivary cortisol^10–12^.

For small and routine invasive procedures, such as port catheter placements, research is limited. Port catheter placement is one of the most frequently performed oncological surgeries. Although it is a small procedure, patients are anxious^13,14^. Zengin and colleagues^15^ conducted a study investigating the effects of a music intervention during port catheter surgery and revealed positive effects of the music on subjective anxiety and pain levels as well as physiological parameters such as heart rate, blood pressure and serum cortisol. The study by Zengin et al.^15^ played the music intervention using a music system in the operating theatre and therefore also the medical staff listened to the music. Consequently, a bias from the staff cannot be omitted. Furthermore, a study by McDaniel et al.^16^ investigated the soothing effects of music during peripherally inserted central catheters and port-a-caths and compared three different conditions: music was played aloud via speakers, music was played via headphones, and no music intervention. The results showed that patients subject to port-a-caths, hearing music via headphones, reported significantly lower anxiety after the procedure compared to the beginning^16^. The finding that only the group, who listened to music via headphones showed a positive effect on subjective anxiety levels, whereas no effect of the music was revealed for the group who listened to music via loudspeakers, can be explained by the fact that the music played via headphones was selected by the patient and the music played via speakers was selected by the radiologist^16^. Additionally, a recent study by Mou and colleagues^17^ showed that music listening during a peripherally inserted central catheter led to decreased subjective anxiety levels, lower diastolic blood pressure levels and lower heart rate levels, whereas systolic blood pressure and respiratory rate were not affected. To the best of our knowledge, the effect of a music intervention during port catheter placement on salivary cortisol levels has not been investigated yet. However, Zengin and colleagues^15^ showed a positive effect on serum cortisol levels and in other medical settings such as caesarean sections soothing effects of music on salivary cortisol levels have been shown^12^. Therefore, the present study included salivary cortisol measures in order to investigate the effect of the music intervention on an easy to administer endocrinological anxiety and stress marker.

An issue regarding the validity of previous studies investigating music interventions in clinical settings is that most studies were not performed blinded^18^, which adds to the concern that many studies in this area are at medium risk of bias^19^. As it is not possible to blind the participants in music studies (unless they are under general anaesthesia), it is desirable to at least blind the personnel. A limited number of studies have performed single-blinded music intervention studies in different surgical settings revealing mixed results^20–23^. A recent study by Cimen and colleagues^24^ conducted a single blinded study investigating the effect of a music intervention during fistula surgery and revealed that the music intervention led to reduced anxiety and pain scores and improved patient satisfaction. To the best of our knowledge, to date in the context of port catheter placements no single-blinded studies have been conducted.

Therefore, the present study examined the influence of an intraoperative music intervention during port catheter placement on anxiety and stress of the patient applying a single-blinded design. We aimed to evaluate the course of anxiety from the preoperative care area until one hour postoperatively and evaluated subjective as well as objective measures of anxiety and stress. Next to the physiological values heart rate and blood pressure, which are commonly investigated in this area of research, we also measured salivary cortisol levels as an objective stress marker. By evaluating the anxiety and stress parameters at different time points, we were interested to investigate the course of anxiety and to explore how the intraoperative music intervention influences anxiety development. Patients were randomly assigned to either the music group (listening to music during the procedure) or the control group (no intervention) and the medical personnel was blind to whether the patient listened to music or not (all patients wore headphones). Anxiety and stress levels were measured by subjective (STAI questionnaire, visual analogue scales) and objective (vital parameters, salivary cortisol) parameters at different time points on the day of the surgery (before, during and after port catheter placement). We hypothesised that the music group will display a greater reduction in anxiety levels from before to the end of the surgery and consequently lower anxiety and stress levels compared to the control group at the end of the surgery.

## Results

### Group characteristics

**Figure 1:**
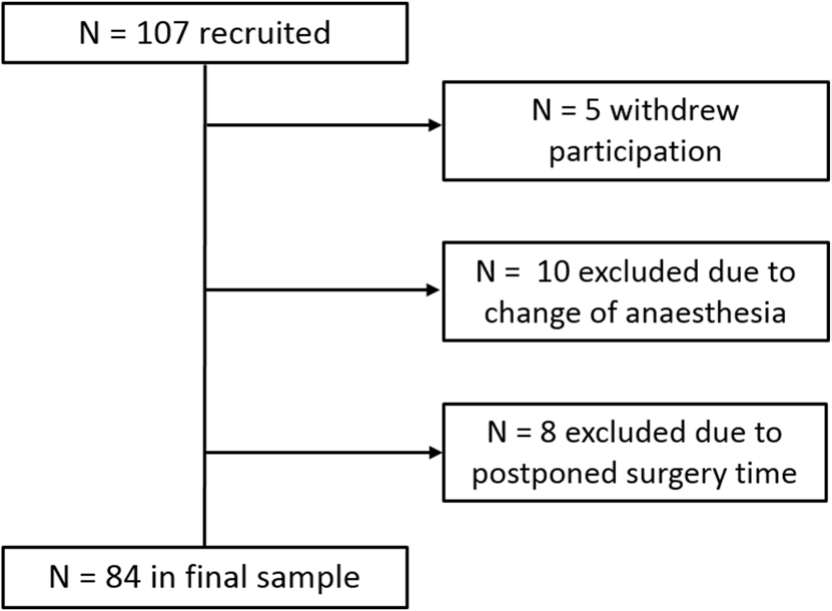
Flow chart of the sample.

From the 107 women recruited, 23 could not be included in the final sample. The sample flow chart and reasons for exclusion are illustrated in Fig. 1.

Therefore, the final sample consisted of 84 female oncology patients with a mean age of 56.6 ± 13.6 years. Most women (N = 59, 70.2%) had a mamma carcinoma whereas 10 women (11.9%) had a cervix carcinoma, eight women (5.9%) had an ovarian carcinoma and seven women (8.3%) suffered from a more seldom cancer type. Forty-four patients were in the music-group and 40 patients were in the control group. In the music group, 15 patients listened to meditation music, 14 to classical music, ten to lounge music and five chose to listen to jazz. The two groups (music vs. control) did not differ regarding age, *t*(82) = 0.35, *p* = 0.728, STAI-Trait, *t*(76) = 1.16, *p* = 0.248 or surgery duration, *t*(75) = 1.36, *p* = 0.117. In the music group the mean age was 56.1 ± 12.9 years, the STAI-Trait was 39.34 ± 9.75 and the mean surgery time 25.9 ± 12.7 min. In the control group the mean age was 57.2 ± 14.4 years, the STAI-Trait score 36.88 ± 9.00 and the mean duration of surgery 22.3 ± 9.8 min. An overview of sample characteristics and the dependent variables is given in Table 1. All patients received 10 ml Xylocaine 1% as local anaesthesia at the beginning of surgery. Additionally, for 78 patients the information of intraoperative systemic analgesics is available (missing data for N = 5). Intraoperatively 76% of the patients (N = 59) received remifentanil (0.1 µg/kg/min). The number of patients, who did or did not receive remifentanil, did not differ between groups, χ^2^ (78) = 0.07, *p* = 0.792. In the music group 30 patients received remifentanil and 9 patients did not and in the control group 29 patients received remifentanil and 10 did not.

**Table 1:** Sample characteristics and overview of dependent variables.

	Music group	Control group	p-value
N	44	40	
Age	56.1 ± 12.9	57.2 ± 14.4	.728
Duration of surgery	25.9 ± 12.7	22.3 ± 9.8	.117
STAI-trait	39.34 ± 9.75	36.88 ± 9.00	.248
STAI-State T1	48.69 ± 10.56	48.77 ± 10.93	
STAI-State T2	38.35 ± 10.64	38.58 ± 10.81	
STAI-State T3	36.73 ± 10.80	37.42 ± 9.66	
VAS-A T1	3.72 ± 2.77	3.77 ± 2.33	
VAS-A T2	1.55 ± 2.67	2.00 ± 1.82	
VAS-A T3	1.74 ± 2.48	1.53 ± 1.65	
VAS satisfaction	8.45 ± 1.77	8.16 ± 2.08	
Systolic BP t1	136.5 ± 26.1	136.7 ± 24.5	
Systolic BP T2	123.3 ± 22.0	132.2 ± 23.9	
Diastolic BP t1	74.7 ± 12.9	72.5 ± 10.3	
Diastolic BP T2	68.7 ± 10.1	69.0 ± 10.5	
Heart rate t1	75.6 ± 12.3	74.0 ± 14.9	
Heart rate T2	73.1 ± 12.2	74.8 ± 14.8	
Cortisol T1	6.74 ± 4.14	8.47 ± 5.36	
Cortisol T2	7.75 ± 9.35	7.12 ± 3.59	
Cortisol T3	4.71 ± 4.00	10.81 ± 13.94	

Displayed are the Mean ± standard deviation. The p-values for the baseline measures are included, highlighting no group differences.

*T1* in the preparation room ~ 15–30 min prior to surgery, *T2* end of the surgery, *T3* 1 h after the surgery, *t1* start of the surgery, *STAI* state-trait-anxiety inventory, *VAS-A* visual analogue scale for anxiety, *BP* blood pressure.

Regarding the musical background of our sample, a music questionnaire revealed that most patients rated music as being very or rather important for them (N = 73) whereas 10 patients indicated that music is rather unimportant. All patients indicated that they listen to music in everyday life. 91% of the sample listen to music when they are happy, 59% when they are sad, 51% when stressed and 33% when they are anxious. From our sample, 69% listen to music while driving, 52% when having breakfast, 64% in order to relax and 18% to go to sleep. There were no group differences regarding the importance and usage of music in everyday life (p-values > 0.219).

### Subjective anxiety measures

A 3 × 2 ANOVA with the STAI-State scores as the dependent variable revealed a significant main effect of *time* (indicating less anxiety after the surgery than before the surgery), *F*(2, 100) = 29.96, *p* < 0.001, whereas the main effect of *group* and the time*group interaction turned out non-significant (p-values > 0.881). The mean STAI-State scores were relatively high at T1 in both groups (48.69 ± 10.56 for music group and 48.77 ± 10.93 for control group), the scores decreased for both groups at T2 and T3 (T2: 38.35 ± 10.64 for music group and 38.58 ± 10.81 for control group; T3: 36.73 ± 10.80 for music group and 37.42 ± 9.66 for control group).

For the 3 × 2 ANOVA with VAS-A as the dependent variable the same pattern occurred. The main effect of *time* was significant, *F*(2, 124) = 19.69, *p* < 0.001 and the main effect of *group* and the time*group interaction were non-significant (p-values > 0.669). At T1 (before the surgery) the mean VAS-A scores were 3.72 ± 2.77 cm in the music group and 3.77 ± 2.33 cm in the control group. The values then decreased resulting in lower levels at T2 (1.55 ± 2.67 cm for music group and 2.00 ± 1.82 cm for the control group) and T3 (1.74 ± 2.48 cm for the music group and 1.53 ± 1.65 cm for the control group).

For the VAS-satisfaction both groups displayed fairly high scores. The music group had a score of 8.45 ± 1.77 cm and the control group 8.16 ± 2.08 cm, and the difference was non-significant, *t* (72) = 0.64, *p* = 0.525, *d* = 0.15 [CI 95% − 0.31, 0.61].

### Physiological measures

**Figure 2:**
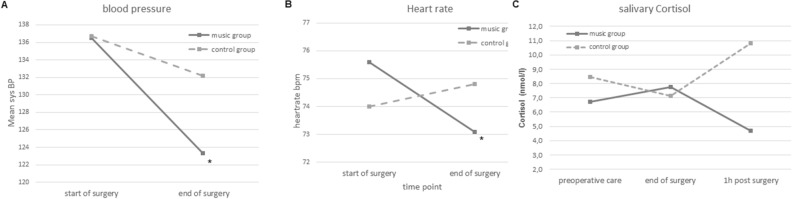
The effect of the music intervention on physiological and endocrinological outcome measures. (**A**) The music group displayed a significant decrease in blood pressure from start to the end of the surgery. (**B**) Heart rate values of the music group reduced significantly from before to the end of the surgery. (**C**) No significant time-group interaction was revealed for salivary cortisol and therefore no post-hoc tests were calculated, however, descriptively the music group shows lover cortisol values 1 h postoperatively. *p < .05.

The 2 × 2 mixed-factorial ANOVA with systolic blood pressure (BP) as the dependent variable revealed a significant effect of *time*, *F*(1, 55) = 17.94, *p* < 0.001, *d* = 0.54 [CI 95% 0.16, 0.91] a non-significant effect of *group*, *F*(1, 55) = 0,57, *p* = 0.452, *d* = 0.20 [CI 95% − 032, 0.072] and a significant time*group interaction, *F*(1, 55) = 4.34, *p* = 0.042, *d* = 0.55 [CI 95% 0.02, 1.08]. The significant time*group interaction indicates that the development of systolic blood pressure differs between groups. In order to clarify the different developments, paired-sample t-tests were calculated. These showed a significant reduction of systolic BP in the music group from before the surgery (136.5 ± 26.1 mmHg) to the end of the surgery (123.3 ± 22.0 mmHg), *t*(28) = 3.92, *p* = 0.002, *d* = 0.69 [CI 95% 0.31, 1.07] (Fig. 2A), whereas in the control group the systolic BP reduction was non-significant (136.7 ± 24.5 mmHg at t1 and 132.2 ± 23.9 mmHg at T2), *t*(27) = 1.85, *p* = 0.150, *d* = 0.35 [CI 95% − 0.02, 0.717].

The same analysis with diastolic BP as the dependent variable showed a significant main effect of *time*, *F*(1, 55) = 22.50, *p* < 0.001, *d* = 0.60 [CI 95% 0.22, 0.97] whereas *group* and the time*group interaction turned out non-significant (p-values > 0.213).

The 2 × 2 ANOVA with heart rate as the dependent variable showed a non-significant main effect of *time*, *F*(1, 63) = 1.07, *p* = 0.305, *d* = 0.14 [CI 95% − 0.21, 0.48] and *group*, *F*(1, 63) = 0.001, *p* = 0.991, *d* = 0.01 [CI 95% − 0.52, 0.52] and a significant time*group interaction, *F*(1, 63) = 4.59, *p* = 0.036, *d* = 0.25 [CI 95% − 0.23, 0.74]. In order to disentangle the significant interaction and clarify the different developments of heart rate in the two groups, paired-samples t-tests were calculated. The results revealed that the music group displayed a significant reduction in heart rate from before (75.6 bpm ± 12.2) to the end (73.1 ± 12.2) of the surgery, *t*(33) = 2.20, *p* = 0.035, *d* = 0.37 [CI 95% 0.03, 0.72] (Fig. 2B), however the control group showed no significant change (74.0 ± 14.9 to 74.8 ± 14.9), *t*(30) = 0.81, *p* = 0.427, *d* = 0.13 [CI 95% − 0.21, 0.48].

For cortisol values as the dependent measure, a 3 × 2 ANOVA revealed a non-significant effect of *time*, *F*(2, 94) = 0.03, *p* = 0.974, a non-significant effect of *group*, *F*(1, 47) = 2.92, *p* = 0.094 and a non-significant time*group interaction, *F*(2, 94) = 2.46, *p* = 0.091. The development of cortisol levels in the two groups is presented in Fig. 2C. Descriptively, the music group displayed lower cortisol levels than the control group 1 h post surgery.

### Exploratory analysis

Bivariate correlations highlight that trait anxiety is positively associated with pre-surgical subjective anxiety measures, such as VAS-A *r* = 0.360, *p* = 0.002 and STAI-State *r* = 0.416, *p* < .001, as well as preoperative salivary cortisol levels *r* = 29, *p* = 0.027. However, when rerunning the above ANOVAs as ANCOVAs with STAI-Trait scores as a covariate, the result patterns did not change.

## Discussion

The present study investigated the effect of an intraoperative music intervention during port catheter placement on patients’ anxiety and stress levels using a single-blinded design. The results revealed favourable effects of the music intervention on systolic blood pressure and heart rate indicating a soothing effect of the music intervention on the autonomic nervous system. The analysis of salivary cortisol levels revealed non-significant results. The hypothesised time*group interactions turned out non-significant. Descriptively, the music group displayed lower cortisol levels than the control group one hour post surgery. More research is necessary in order to investigate whether music listening during port-catheter placement leads to significant reductions in salivary cortisol levels, as the presented results are inconclusive. Regarding subjective values, the effects of the music intervention also turned out non-significant. An interesting exploratory analysis showed that anxiety trait correlates with preoperative subjective anxiety measures as well as salivary cortisol levels, highlighting that trait anxiety is an interesting factor in the context of surgical anxiety.

### Main findings

The results revealed that an intraoperative music intervention during port catheter placements led to significant reductions in heart rate and blood pressure in the music group whereas the control group did not show significant changes on these variables, indicating an anxiety-reducing effect of the music intervention. These results are in accordance with previous studies also highlighting soothing effects of music interventions during different clinical settings on vital signs^6,8,9,15,17^. According to Bernardi et al.^25^, the positive effect of music on autonomic functions might be unfolded due to a synchronization of autonomic responses. It is hypothesised that the endogenous rhythms of the nervous system might entrain to the relaxing rhythms of the music. Additionally, a decrease in heart rate is an indicator for the dominating role of parasympathetic activity. As activations of the autonomic nervous system can be provoked in dependence of emotions evoked by music, Koelsch and Jäncke^26^ suggested that relaxing music leads to lower heart rate than exciting music due to different grades of emotional arousal.

The analysis investigating the effect of the music intervention on salivary cortisol revealed non-significant results. As the time*group interaction turned out non-significant, no post-hoc tests were calculated. However descriptively, the data showed that the music group displayed lower salivary cortisol levels compared to the control group two hours after the surgery, which might suggest a soothing effect of the music intervention on the neuroendocrine stress system. However, due to the non-significant interaction, the results of the music intervention on salivary cortisol remain inconclusive. Previous studies in the context of caesarean sections and elective total hip joint replacement reported significant lower salivary cortisol levels in the music compared to the control group^11,12^. In the context of port catheter placements a previous study showed a positive effect of music on serum cortisol^15^. More research is needed to investigate whether salivary cortisol can be a sufficient measure to evaluate the effect of music on endocrinological anxiety levels in small invasive procedures such as port catheter placement. It would be intriguing if future studies would reveal significant effects of music interventions on salivary cortisol levels in this context as no invasive method needs to be applied and therefore it is favourable for the patient. Descriptively, the two groups showed a different anxiety response one hour after the surgery, which can be explained by the well-established approximate 15–30 min latency of cortisol detection in saliva^27^. In regard to the development of cortisol responses, Khoo and colleagues^28^ revealed that for mild surgeries, the peak cortisol levels can be expected even later—up to two hours postoperatively. In this respect, it would be interesting to investigate whether an intraoperative music intervention during port catheter surgery could maybe have an effect on cortisol levels measured at the proposed peak of salivary cortisol two hours postoperatively. In order to investigate this, a follow-up study measuring salivary cortisol one and two hours postoperatively would be desirable.

Regarding subjective anxiety measures, the present study revealed no significant effects of the music intervention on neither the STAI-State nor the VAS for anxiety. This is in contrast to previous studies, which showed a positive effect of music intervention on these dependent variables in the setting of port catheter surgery^15,17^ and other surgical settings^7,12,29^.

### Interpretation

It was somehow surprising that we revealed significant positive effects of the music intervention on physiological parameters, whereas the effect on subjective factors turned out non-significant. An extensive recently published review on the effects of music intervention on stress conducted two separate meta-analyses focussing on the impact of the music on physiological and psychological measures independently^30^. This emphasizes that although the physiological and psychological reactions of stress and anxiety usually relate to one another the influence of music interventions may be specific to different aspects^30^. Furthermore, we applied a single-blinded design in the present study. To the best of our knowledge, this is the first single-blinded study in the context of port catheter surgery and maybe this is one decisive factor why our results are in contrast to previous studies, which reported that music interventions during port catheter surgery also affect subjective anxiety measures^15,17^. This suggests a bias by the medical staff if group allocation is known or that the music intervention could have a calming effect on the staff members themselves^31^, which is then passed to the patient.

Furthermore, it may be hypothesised that the control group of the present study profited by wearing headphones and being shielded from the noises in the operating theatre and therefore the subjective anxiety measures were also influenced. This could explain why no differences to the music group were revealed regarding subjective anxiety measures. This assumption should be tested in a follow up study by comparing three groups: music intervention through headphones, wearing headphones without auditory intervention and one group with no headphones or intervention. Another aspect, which should be considered here, is the length of the music intervention. In the present study, the music intervention was only played during the surgery, which was on average 23 min long (range: 12–40 min). In comparison to other studies in the context of port operations, which either played the music 30 min before the operation as well as during surgery^15^ or for at least 30 min during the surgery^17^, our music intervention is relatively short. It can be hypothesised that the intervention in the present study was not long enough to affect subjective parameters or salivary cortisol. In this context, the review of de Witte and colleagues^30^ stresses that the relationship of the (optimal/minimal) duration of the music intervention and the effect of stress and anxiety reduction is not clear and should be subject to future research. In addition, the choice of which music is used in the studies may be important regarding the different effects of the music intervention on subjective measures. For example, in the single blinded study by Cimen et al.^24^ the patients listened to the music of their choice and here a soothing effect was revealed for physiological and subjective anxiety measures. In the present study, patients could choose from four genres, but the music itself was selected by the study team. It would be valuable to compare the different methods of music selection within one study.

Furthermore, a review of our group comparing the effects if preoperative, intraoperative and postoperative music interventions on pain perception concluded that especially preoperative music interventions lead to reduced pain perception^32^. In this context, it would be of interest to conduct the present study again and apply the music intervention in the preoperative setting.

Although, studies investigating the mechanisms behind the therapeutic and anxiety reducing effect of music are sparse, it is proposed that physiological changes evoked by listening to music might emerge due to the links of brain regions of the limbic system to endocrine functions and autonomic mechanisms^33^. In this respect, it is suggested that the music suppresses the activation of the sympathetic nervous system which then leads to a decrease in adrenergic activity^19^. The effect of music interventions might also be based on the distraction from unpleasant emotions^33^, improving mood and evoking positive memories^34^.

The study highlights that the music intervention led to a significant decrease in heart rate and blood pressure. These are encouraging results. However, the clinical relevance of the observed results are under debate and additional studies are needed to further investigate this. In this regard a systematic review on the quality of available music intervention studies discusses the importance of high quality studies which give sufficient information regarding the music intervention applied in order to improve the clinical relevance of music interventions^35^.

Additionally, our study revealed that STAI-Trait scores, measuring anxiety as a personality trait, positively correlated with preoperative subjective anxiety scores. The finding that higher anxiety trait scores are associated with higher preoperative anxiety levels has already been shown in other clinical contexts, such as caesarean sections^36^. It seems that anxiety trait scores play an important factor regarding anxiety levels experienced during surgery. Even though, the result patterns regarding the effects of music on subjective measures did not change when controlling for STAI-Trait scores, it would be of great interest to perform a music intervention study with high anxiety trait patients (screened with the STAI-Trait) and investigate whether highly anxious patients would profit more from a music intervention than less anxious patients would. This could be especially interesting for oncology patients, where anxiety symptoms often accompany the course of disease^37^.

The soothing effect of the music intervention during port catheter placement in analgosedation on physiological anxiety and stress parameters should encourage the medical staff to offer this easy-to-implement and low-cost intervention to oncology patients, in order to make this small invasive procedure more pleasant for the patient. The anxiety and stress-reducing effect of music is important for oncology care as it might improve the accessibility and motivation of patients^20^, which in turn leads to an improved cooperation between patient and staff. By this, the positive effect of music could also improve the willingness of patients to continue important oncology treatments.

### Strengths and limitations

This study was performed at a single centre and furthermore only female oncology patients were included, which limits the generalisation of the study results. Additionally, some patients (N = 5) withdrew participation on the day of the surgery as they felt too unsettle before the operation and 10 patients had to be excluded as the choice of anaesthesia was changed and they were not awake during the procedure. This is a limitation to that extend, that maybe the high anxious patients did not take part in the present study, however it is possible, that especially these patients may benefit from a music intervention. There are data from a study in the context of intracardiac catheterization demonstrating, that patients with high psychological strains (i.e. very anxious patients) benefited more from the music intervention than patients in the low psychological strains group^38^. All in all, the present sample was not very anxious before the study as highlighted by relatively low VAS-A scores of 3.72 cm for the music group and 3.77 cm for the control group from a possible maximum anxiety of 10 cm. This may also be the reason why no effects of the music intervention were found on subjective anxiety measures as well as salivary cortisol. Furthermore, for the determination of saliva cortisol missing values were present, as too little saliva was collected which made the analysis impossible. For these missing values, we applied a single imputation method by inserting the overall median, as this is a simple to implement method. It avoids loss in power as the participants with missing values do not need to be excluded. Although single imputation is a common method^39–41^, we acknowledge that other more complex methods are available, such as multiple imputation, which account for the uncertainty and range of the inserted values. Regarding the results of salivary cortisol future research is needed in order to reveal support for the positive effect of music intervention on endocrinological stress levels of patients. Despite these limitations, we believe that the single-blinded design, as well as the evaluation of subjective and objective anxiety and stress measures, also including salivary cortisol, at three different time measures present meaningful results, which should encourage further research. Additionally, as the focus of the present study was on patients’ anxiety, we did not investigate the effect of music on pain management and perception, but this is something we want to examine in the near future. As previous studies have shown that listening to music during surgery also leads to reduced pain levels^15,42^, it would be interesting to investigate this in the context of port catheter placement in more detail and using a single-blinded design in order to replicate and expand the reported positive effect of music by Zengin and colleagues^15^ in the context of port catheter placement.

## Conclusion

Taken together the study demonstrates a positive influence of a music intervention in the setting of port catheter placement on physiological anxiety measures. Therefore, music can be considered as a risk-free and low cost addition to the clinical routine in order to reduce physiological anxiety measures such as heart rate and blood pressure in patients receiving port catheter placement.

## Methods

### Participants

From December 2015 to December 2019 gynaecological oncology patients, who had an indication for port catheter placement at the Clinic for Gynecology and Obstetrics at the University Hospital in Düsseldorf were offered participation. In total, 107 women agreed to take part in this single-blinded, randomised controlled two-armed trial with a 1:1 allocation. Inclusion criteria were that women had an indication for a planned placement of a port catheter under analgosedation to a degree that did not necessitate respiratory support, and adequate German language comprehension in order to answer the questionnaires. Exclusion criteria were patients requiring general anaesthesia as well as known anxiety disorders or other severe psychiatric illnesses. An a-priori power analysis for sample-size estimation was calculated based on the primary outcome measure *salivary cortisol* using G*Power (www.gpower.hhu.de)^43^. Given an expected small to medium effect size of f = 0.25, a power of 80% and an alpha-error of 0.05 based on a mixed-factorial 3 × 2 ANOVA the required sample-size was 86 participants. Secondary outcomes were heart rate, systolic and diastolic blood pressure, STAI-State and VAS-A. In the trial registry, also the outcome measure skin resistance was defined. However, this outcome measure was then not evaluated in the present study due to the impracticability of the measure.

The study was prospectively approved by the ethics committee of the Medical Department of the Heinrich- Heine-University in Düsseldorf (No. 5209) on 17.09.2015 and prospectively registered at the Deutsche Register Klinischer Studien (DRKS00009036) on 28.09.2015. The research was conducted in accordance with the Helsinki Declaration and the manuscript conforms to the CONSORT 2010 guidelines.

### Material and measures

In order to determine the primary outcome *cortisol levels* as a measure of the endocrine stress response system, saliva samples were collected. For this, patients were asked to insalivate cotton swabs. Cortisol levels are a biomarker for the activation of the hypothalamus–pituitary–adrenal axis^44^. The saliva samples were kept frozen at − 18 °C until analysed at the DresdenLab following the methods described elsewhere^45^. Inter- and intraassay variances were below 9%.

As physiological measures of anxiety and stress vital signs such as heart rate and blood pressure were documented by a member of the team at predetermined time points. The outcome measures for heart rate and blood pressure were at the beginning and the end of the surgery.

To measure the degree of subjective anxiety, the German version of the State-Trait Anxiety Inventory (STAI), developed by Laux et al.^46^ was used. The STAI is a valid and reliable questionnaire with 40 items. Twenty items each refer to State and Trait anxiety respectively. Whereas the Trait questionnaire evaluates anxiety as a personality trait, the State part examines situational anxiety. Ratings are done on 4-point Likert scales. Summation leads to scores between 20 and 80 for each part, with higher scores indicating higher State or Trait anxiety.

Furthermore, visual analogue scales (VAS) were used as an instrument to measure subjective anxiety levels^47^. Ratings are done by marking a point on a 10.0 cm horizontal line that corresponds to the perceived degree of anxiety. Possible scores can be reached on a continuum from 0 (no anxiety) to 10 (maximal anxiety). For evaluation, the distance (in cm) from point 0 to the marked point is measured. Higher scores indicate more anxiety. We also included a visual analogue scale to measure the satisfaction with the surgery.

In order to evaluate the musical background of the patients a self-created questionnaire (see Supplementary Information) was used. We asked them how important music was in their daily life on a 4 point scale with the options 1 = very important, 2 = rather important, 3 = rather unimportant and 4 = very unimportant. We also asked them whether they listen to music in specific everyday situations (i.e. when driving a car, during breakfast, when going to sleep) or when feeling a specific emotion (i.e. happy, sad, anxious, stressed).

### Music intervention

Four music lists with different types of music (jazz music, classical music, lounge music, meditation music) were provided by the researchers for the intervention. Each patient in the music group selected one type of music genre she wanted to listen to during surgery. Music was carefully selected following recommendations described elsewhere^48^. To prevent confounding effects caused by emotion-evoking texts, only instrumental music without lyrics was used. All participants wore noise-cancelling supra-aural headphones (Bose), connected to an mp3-player (Ipod nano, Apple). Only the members of the music group received music. In order to blind the medical staff, also the members of the control group wore headphones that were connected to the mp3-player. To ensure blinding throughout surgery, all participants were asked not to give information about their group assignment to the medical staff in the operating theatre.

### Procedure

Participants were recruited on the day of the patients’ preadmission appointment approximately three days before the surgery. After signing the consent form, they were asked to fill in the STAI-Trait questionnaire. Participants were then randomized (computer-assisted) into the music or control group but were not yet told about their group allocation. Randomisation was done using a computer-based controlled randomisation with the allocation ratio 1:1 (no block randomisation). As soon as the patient was included in the study, patient information was entered into the randomisation list and the excel sheet revealed group allocation (music vs. control group). The random allocation list was generated by the author P.H. and a research assistant enrolled participants and assigned them to an intervention arm.

**Figure 3:**
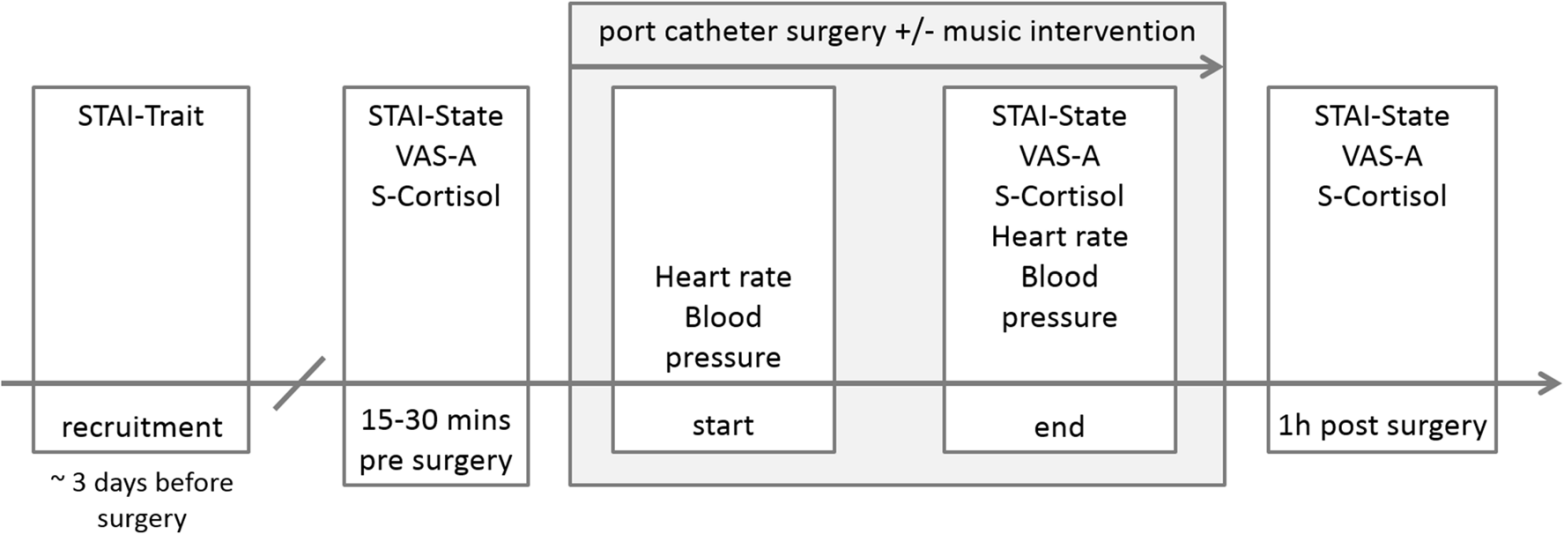
Procedure of the study highlighting which measures were obtained at the different time points.

On the day of the surgery, about 15 min before the surgery the participants were visited in the preoperative care area by a member of the research team. At this measuring time point the first saliva sample was taken. In addition, the participants answered the first STAI-State questionnaire and marked their perceived anxiety on a VAS. Then they were informed about their group assignment. The members of the music group chose one type of music they wanted to listen to during the surgery. The volume of the music was individually adjusted to a comfortable level. In the operating theatre, just before the start of the operation, heart rate and blood pressure were documented by a team member and the head phones were placed on the participants head. The music started in the music group. The next measuring time point was at the end of the surgery. While the skin suture was done, the music intervention stopped, the items of the STAI and in the VAS were answered again by the participant and a second saliva sample was taken. Furthermore, heart rate and blood pressure were documented by the research assistant. In the operating theatre all medical staff were blind to whether the patient listened to music or not. In order to blind the medical team, all patients (independent of group allocation) wore head phones and the patients were asked not to mention whether they were listening to music or not. The research assistant who accompanied the patient in the operating theatre and administered the questionnaires was not blinded. At the last measuring time point, about one hour post-surgery, the patients were asked to fill in the State questionnaire and the VAS for anxiety again and a third saliva sample was taken. Patients were also asked to rate on a VAS how satisfied they were with the procedure and in order to evaluate their musical background we asked them how important music is for them in daily life and in which situations they usually listen to music. The procedure is illustrated in Fig. 3. The patients received a small thank you gift.

### Statistical analysis

In order to compare baseline group characteristics independent sample t-tests with group (music vs. control) as the between-group factor and age, STAI-Trait scores and surgery duration as the dependent factors respectively were calculated. Chi-square tests were applied to investigate group differences regarding the importance and use of music in everyday life.

Between group differences for heart rate and blood pressure measured at the beginning and end of the surgery were explored with two 2 × 2 mixed-factorial ANOVAs with the between-subject factor group (music group vs. control group) and the within-subject factor time (start and end of the surgery) and heart rate and blood pressure respectively as the dependent variable were calculated. In order to clarify the significant group*time interactions and check for the changes within group from beginning to end of the surgery paired-samples t-tests were calculated.

For salivary cortisol, STAI-State and VAS for anxiety measures were taken at three time points. Therefore, 3 × 2 mixed-factorial ANOVAs with the between-subject factor group (music group vs. control group) and the within-subject factor time (preoperative area, end of the surgery and 1 h post-surgery) were applied.

For the cortisol values, the following rule was applied for missing values: if one out of three saliva measurements was missing (mostly due to too little amount of saliva) in one patient, we replaced the missing value with the overall sample median. This is a conservative and common method and was done for 15 patients.

In order to investigate whether trait anxiety influences preoperative anxiety levels, an exploratory analysis was carried out by calculating bivariate correlations between the STAI-Trait scores and preoperative anxiety measures (STAI-State, VAS-A and salivary cortisol). Bonferroni-Holm corrections were applied to avoid Type I errors.

### Ethical approval

The study was prospectively approved by the ethics committee of the Medical Department of the Heinrich-Heine-University in Düsseldorf (No. 5209) on 17.09.2015 and registered at the Deutsche Register Klinischer Studien (DRKS00009036). All participants gave informed written consent prior to participation.

## Supplementary Information

Supplementary Information

## Data Availability

The datasets used and analysed during the current study are available from the corresponding author on reasonable request.
