# Supplementary material for: The effects of a music intervention during port catheter placement on anxiety and stress
Source: Sci Rep. 2021 Mar 11;11:5807. doi: 10.1038/s41598-021-85139-z (PMC7970967; doi:10.1038/s41598-021-85139-z)
Supplement: Supplementary file 1 — Supplementary Information [file 41598_2021_85139_MOESM1_ESM.pdf]

Supplementary Information

Manuscript Title: A single-blinded randomised controlled trial investigating the influence of a music intervention during port catheter placement on anxiety and stress

Authors; Nora K. Schaal, Johanna Brückner, Oliver T. Wolf, Eugen Ruckhäberle, Tanja Fehm, Philip Hepp

Music Questionnaire

1. How important is music for you in everyday life?
   ☐ very important
   ☐ rather important
   ☐ rather unimportant
   ☐ very unimportant

2. In which emotional states do you listen to music? (multiple select)
   ☐ when I feel good
   ☐ when I feel sad
   ☐ when I feel stressed
   ☐ when I am anxious

3. In which Situation do you hear music? (multiple select)
   ☐ when driving the car
   ☐ during breakfast
   ☐ in order to relax
   ☐ when going to sleep
